# Usp31 Promotes Cardiomyocyte Injury Under Ischemic Stress and Is Inhibited by Sodium Tanshinone IIA Sulfonate

**DOI:** 10.3390/biomedicines14081812

**Published:** 2026-08-12

**Authors:** Zikan Zhong, Chenyang Jin, Xudong Li, Longzhe Gao, Yutong Ye, Lin Liang, Tong Wei, Xiaofeng Lu, Jun Li, Shaowen Liu, Songwen Chen, Juan Xu

**Affiliations:** Department of Cardiology, Shanghai General Hospital, Shanghai Jiao Tong University School of Medicine, No. 100 Haining Road, Shanghai 200080, China; zzk991226@163.com (Z.Z.); yang1026abc@163.com (C.J.); lxd2024@sjtu.edu.cn (X.L.); m18516012301@163.com (L.G.); yehyutong@yeah.net (Y.Y.); lianglin_shirley@163.com (L.L.); wei_tong_1004@163.com (T.W.); luxiaofeng@sjtu.edu.cn (X.L.); junli@shsmu.edu.cn (J.L.)

**Keywords:** Usp31, deubiquitination, NF-κB signaling, oxygen-glucose deprivation/reoxygenation, sodium tanshinone IIA sulfonate

## Abstract

**Background/Objectives:** Ischemia-reperfusion injury (IRI) contributes significantly to cardiomyocyte death following myocardial infarction, largely through sustained inflammatory signaling. This study aimed to identify stress-responsive deubiquitinating enzymes involved in cardiomyocyte injury under ischemic stress. **Methods:** HL-1 cardiomyocytes were subjected to oxygen-glucose deprivation and reoxygenation (OGD/R), followed by transcriptomic analysis, genetic manipulation of Usp31, biochemical assessment of p65 ubiquitination, NF-κB reporter assays, and in vitro deubiquitinase activity assays using sodium tanshinone IIA sulfonate (STS). **Results:** Usp31 was persistently upregulated in HL-1 cardiomyocytes after OGD/R. Depletion of Usp31 improved cell viability and reduced lactate dehydrogenase (LDH) release under OGD/R conditions. Mechanistically, Usp31 stabilized p65 and supported NF-κB activation, whereas Usp31 deficiency increased p65 ubiquitination and limited NF-κB transcriptional activity. STS inhibited recombinant Usp31 enzymatic activity under the experimental conditions tested and reduced Usp31-associated NF-κB activation and cardiomyocyte injury in cellular assays. **Conclusions:** These findings identify Usp31 as a candidate regulator of NF-κB-associated inflammatory responses in cardiomyocytes and support STS as a pharmacological modulator of Usp31-associated signaling with potential relevance to myocardial ischemic injury.

## 1. Introduction

Myocardial ischemia-reperfusion injury (IRI) remains a major clinical challenge and a leading cause of cardiomyocyte death following acute myocardial infarction and subsequent reperfusion therapy [1]. While the restoration of blood flow is essential for tissue salvage, the reoxygenation phase paradoxically triggers a secondary wave of cellular injury mediated by oxidative stress, calcium overload, and inflammatory signaling cascades [2,3]. Among these, sustained activation of the nuclear factor kappa-light-chain-enhancer of activated B cells (NF-κB) pathway plays a central role in amplifying inflammatory responses and exacerbating myocardial damage [4].

The NF-κB signaling pathway is tightly regulated at multiple levels, including ubiquitin-dependent degradation of its key components [5]. Deubiquitinating enzymes (DUBs) have recently emerged as crucial modulators of NF-κB activity by controlling the stability of upstream adaptor proteins and transcription factors [6,7,8]. However, the identity of specific DUBs that drive persistent NF-κB activation in cardiomyocytes under ischemic stress remains poorly understood.

In this study, we performed transcriptomic profiling of HL-1 cardiomyocytes subjected to oxygen-glucose deprivation and reoxygenation (OGD/R), a widely used in vitro model that recapitulates key features of myocardial IRI [9,10]. Our analysis revealed that Usp31, also known as Usp48 [11], a member of the ubiquitin-specific protease family [12], was continuously upregulated during the transition from OGD to OGD/R. Usp31 has previously been implicated in NF-κB regulation through stabilization of TRAF2 and p65 in non-cardiac contexts [11,13], but its role in myocardial stress has not been explored.

In this study, we aimed to investigate the role and regulatory mechanism of Usp31 in cardiomyocyte responses to ischemic stress. Using an oxygen-glucose deprivation/reoxygenation (OGD/R) model in HL-1 cardiomyocytes, combined with transcriptomic analysis, genetic manipulation, and biochemical approaches, we examined whether Usp31 contributes to NF-κB signaling regulation during ischemic injury. Furthermore, we explored whether sodium tanshinone IIA sulfonate (STS), a clinically used cardiovascular agent [14,15], modulates Usp31-associated signaling and attenuates OGD/R-induced cardiomyocyte injury. Our findings identify Usp31 as a candidate regulatory component involved in NF-κB-mediated inflammatory responses during ischemic stress and provide insights into the potential mechanisms underlying the pharmacological effects of STS.

## 2. Materials and Methods

### 2.1. Cell Culture and OGD/R Treatment

Mouse HL-1 cardiomyocytes (MilliporeSigma, Burlington, MA, USA) were cultured in Claycomb medium (Sigma-Aldrich, St. Louis, MO, USA) supplemented with 10% fetal bovine serum (Gibco, Thermo Fisher Scientific, Waltham, MA, USA), 2 mM L-glutamine (Gibco, Thermo Fisher Scientific, Waltham, MA, USA), 0.1 mM norepinephrine (Sigma-Aldrich, St. Louis, MO, USA), and penicillin/streptomycin (Gibco, Thermo Fisher Scientific, Waltham, MA, USA). For oxygen-glucose deprivation (OGD), cells were incubated in glucose-free DMEM (Gibco, Thermo Fisher Scientific, Waltham, MA, USA) in a hypoxia chamber (1% O_2_, 5% CO_2_) for 6 h. Reoxygenation was achieved by returning cells to normoxic conditions in complete medium for an additional 6 h. For the physiologic oxygen-based protocol, cells were maintained under 5% O_2_ before OGD and reoxygenated under 5% O_2_ with glucose replenishment, while OGD was performed under 1% O_2_ in glucose-free medium for 6 h. For OGD/R experiments, cells were incubated in glucose-free DMEM under hypoxic conditions (1% O_2_, 5% CO_2_) for 6 h, followed by reoxygenation in complete medium for an additional 6 h. This 6 h OGD/6 h reoxygenation protocol was selected based on preliminary optimization and provided a reproducible injury model while preserving sufficient viable cells for downstream mechanistic analyses.

### 2.2. CRISPR/Cas9-Mediated Gene Editing

Usp31 was knocked out in HL-1 cardiomyocytes using a lentiviral CRISPR/Cas9 system. Two single-guide RNAs (sgRNAs) targeting exon regions of mouse Usp31 were designed and cloned individually into the lentiCRISPR v2 vector (Addgene #52961; Addgene, Watertown, MA, USA), which co-expresses Cas9 and a puromycin resistance cassette. The sgRNA sequences were as follows: sgRNA #1: 5′-AGGTGTATAATAAGCACATC-3′; sgRNA #2: 5′-CAAACCTGGATGCTGACAGT-3′.

Lentiviral particles were produced in HEK293T cells by co-transfecting the sgRNA-containing lentiCRISPR_v2 plasmid with the packaging plasmid Δ8.9 and the envelope plasmid pMDG using Lipofectamine 3000 (Invitrogen, Carlsbad, CA, USA). Viral supernatants were collected 24 h after transfection, filtered through a 0.45 μm membrane, and used to infect HL-1 cells in the presence of 8 μg/mL polybrene. Transduced cells were selected with 2 μg/mL puromycin for 5-7 days. Single-cell clones were isolated by limiting dilution and screened for Usp31 knockout by immunoblotting.

### 2.3. Usp31 Overexpression

The full-length mouse Usp31 coding sequence was amplified by PCR and cloned into the pLVX-Puro lentiviral expression vector (Clontech, Mountain View, CA, USA), with a C-terminal 3×Flag tag for detection and immunoprecipitation. The integrity of the construct was verified by Sanger sequencing. Lentiviral particles were produced in HEK293T cells by co-transfecting pLVX-Usp31-3×Flag with the packaging and envelope plasmids described above, and HL-1 cells were transduced as described above. Stably infected cells were selected with 2 μg/mL puromycin for 5-7 days. Overexpression of Flag-tagged Usp31 was confirmed by immunoblotting using anti-Flag. Control cells were transduced with the corresponding empty pLVX vector and subjected to identical lentiviral infection and puromycin selection procedures.

### 2.4. RNA Sequencing and Analysis

Total RNA was extracted from HL-1 cardiomyocytes using TRIzol reagent (Invitrogen, Carlsbad, CA, USA) according to the manufacturer’s protocol. Cells were cultured under three different conditions: normoxia control, oxygen-glucose deprivation for 6 h (OGD), and OGD followed by 6 h of reoxygenation (OGD/R). Each group included two biological replicates. RNA concentration and integrity were assessed using a NanoDrop spectrophotometer (Thermo Fisher Scientific, Waltham, MA, USA) and Agilent Bioanalyzer 2100 (Agilent Technologies, Santa Clara, CA, USA). High-quality RNA samples (RIN > 8.0) were used for library construction and sequencing on the Illumina HiSeq platform (Illumina, San Diego, CA, USA) with 150 bp paired-end reads. Raw sequencing data were processed for quality control using FastQC (version 0.11.9) and trimmed with Trimmomatic (version 0.39). Clean reads were mapped to the mouse genome (GRCm38/mm10) using HISAT2 (version 2.2.1). Transcript abundance was quantified with featureCounts (Subread package, version 2.0.1). Genes with low read counts were filtered before differential expression analysis. Differential expression analysis was performed using DESeq2 (version 1.30.1), and *p* values were adjusted for multiple testing using the Benjamini-Hochberg method to control the false discovery rate (FDR). Genes with an absolute fold change ≥ 2 and an FDR-adjusted *p* value ≤ 0.05 were considered differentially expressed. Principal component analysis (PCA) was performed using normalized expression values to evaluate the global transcriptomic relationships among experimental groups.

For validation, reverse transcription was performed using PrimeScript RT reagent kit (Takara Bio, Kusatsu, Shiga, Japan), and qRT-PCR was conducted using SYBR Green Master Mix (Takara Bio, Kusatsu, Shiga, Japan) on a QuantStudio 6 Flex Real-Time PCR system (Applied Biosystems, Thermo Fisher Scientific, Waltham, MA, USA). Gene expression levels were normalized to *Gapdh*, and relative expression was calculated using the 2^−ΔΔCt^ method. Each reaction was run in triplicate. Primer sequences are listed in Table S1.

### 2.5. Cell Viability and LDH Release Assays

Cell viability was measured using the Cell Counting Kit-8 (CCK-8; Dojindo Molecular Technologies, Kumamoto, Japan) following the manufacturer’s instructions. Usp31-knockout and wild-type HL-1 cardiomyocytes were seeded in 96-well plates at a density of 5 × 10^3^ cells per well and cultured overnight. Cells were subjected to either normal control or oxygen-glucose deprivation for 6 h followed by 6 h of reoxygenation (OGD/R). After treatment, 10 μL of CCK-8 solution was added to each well, and plates were incubated at 37 °C for 2 h. Absorbance at 450 nm was measured using a microplate reader (BioTek Instruments, Winooski, VT, USA). Cell viability was expressed as relative absorbance normalized to the control group.

Lactate dehydrogenase (LDH) release into the culture supernatant was assessed as an indicator of plasma membrane damage using a commercial LDH Cytotoxicity Assay Kit (Beyotime Biotechnology, Shanghai, China). Briefly, culture medium was collected from each well after OGD/R or normal control treatment. Equal volumes of reaction solution were added to the supernatant in a 96-well plate and incubated for 30 min at room temperature in the dark. Absorbance was measured at 490 nm using a microplate reader (BioTek Instruments, Winooski, VT, USA). LDH release was calculated as a percentage of total LDH, based on maximum release controls. All assays were performed in triplicate and repeated independently at least three times.

### 2.6. Luciferase Reporter Assays

NF-κB transcriptional activity was assessed using a dual-luciferase reporter system. HL-1 cells with or without Usp31 knockout or Usp31 overexpression were co-transfected with 200 ng of the pNF-κB-Luc firefly luciferase reporter plasmid (Promega, Madison, WI, USA) and 20 ng of the pRL-TK Renilla control plasmid using Lipofectamine 3000 (Invitrogen, Carlsbad, CA, USA). For rescue experiments, Usp31 was re-expressed in Usp31-deficient cells as indicated. After 24 h of transfection, cells were subjected to either normoxia or OGD for 6 h followed by reoxygenation for 6 h. In indicated groups, sodium tanshinone IIA sulfonate (STS) was added during the reoxygenation phase. Luciferase activity was measured using the Dual-Luciferase Reporter Assay System (Promega, Madison, WI, USA), and luminescence was recorded on a Synergy H1 microplate reader (BioTek Instruments, Winooski, VT, USA). Relative NF-κB activity was expressed as the ratio of firefly to Renilla signals. All measurements were performed in triplicate and repeated in at least three independent experiments.

### 2.7. Immunoblotting and Co-Immunoprecipitation

Cells were lysed in ice-cold RIPA buffer (50 mM Tris-HCl, pH 7.4; 150 mM NaCl; 1% NP-40; 0.1% SDS; 0.5% sodium deoxycholate) supplemented with protease and phosphatase inhibitors. For co-immunoprecipitation, equal amounts of total protein were incubated overnight at 4 °C with anti-Flag antibody (for USP31-Flag constructs) or anti-p65 antibody, followed by incubation with Protein A/G agarose beads for 2 h at 4 °C. Beads were washed thoroughly with lysis buffer, and bound proteins were eluted by boiling in SDS loading buffer for 10 min.

For immunoblotting, total protein lysates or immunoprecipitated samples were resolved by SDS-PAGE and transferred onto nitrocellulose (NC) membranes. Membranes were blocked in 5% non-fat milk prepared in TBST (20 mM Tris-HCl, 150 mM NaCl, 0.1% Tween-20) for 1 h at room temperature, then incubated with primary antibodies overnight at 4 °C. After washing, membranes were incubated with horseradish peroxidase (HRP)-conjugated secondary antibodies for 1 h at room temperature. Signal detection was performed using enhanced chemiluminescence (ECL), and bands were visualized using a Bio-Rad imaging system (Bio-Rad Laboratories, Hercules, CA, USA). Primary antibodies used for immunoblotting and immunoprecipitation included anti-Usp31 (17495-1-AP; Proteintech, Wuhan, China), anti-p65 (#8242; Cell Signaling Technology, Danvers, MA, USA), anti-ubiquitin (ab7254; Abcam, Cambridge, UK), anti-Flag M2 (F1804; Sigma-Aldrich, St. Louis, MO, USA), anti-Bnip3 (12396-1-AP; Proteintech, Wuhan, China), anti-Hsp90b1 (11520-1-AP; Proteintech, Wuhan, China), anti-p50 (14220-1-AP; Proteintech, Wuhan, China), anti-TRAF2 (ab24192; Abcam, Cambridge, UK), and anti-ubiquitin (#3936; Cell Signaling Technology, Danvers, MA, USA). Appropriate HRP-conjugated secondary antibodies were obtained from Cell Signaling Technology (Danvers, MA, USA). Antibodies were diluted in TBST containing 5% BSA or milk as indicated. Western blot experiments were performed using independently prepared biological samples and repeated at least three times (n = 3). Representative immunoblots are shown. Where proteins were detected on separate membranes, the membranes were prepared from the same batch of biological samples, equal amounts of total protein were loaded, and the corresponding loading controls were included. Molecular weight markers were added to the revised immunoblot figures.

### 2.8. In Vitro DUB Activity Assay

Recombinant mouse USP31 was expressed in *E. coli* BL21 (DE3) cells using the pGEX-6P-1 vector (Cytiva, Marlborough, MA, USA) and purified by glutathione-Sepharose 4B affinity chromatography (Cytiva, Marlborough, MA, USA) under native conditions. The GST-tagged protein was eluted with 10 mM reduced glutathione in Tris-HCl buffer (50 mM Tris, pH 8.0; 150 mM NaCl) and dialyzed to remove excess glutathione. Protein concentration and purity were assessed by BCA assay and SDS-PAGE, respectively.

Deubiquitinase activity was measured using the fluorogenic substrate Ub-Rho110 (Keygentec, Hefei, China). The reaction mixture (total volume of 50 μL) contained 100 nM purified USP31 protein and 250 nM Ub-Rho110 in assay buffer (50 mM Tris-HCl, pH 7.5; 5 mM DTT; 0.01% Tween-20). Reactions were performed in black 96-well plates at 37 °C, and fluorescence was measured every 2 min for 30 min using a Synergy H1 microplate reader (BioTek Instruments, Winooski, VT, USA; Ex 485 nm, Em 535 nm).

To assess enzymatic inhibition, Usp31 was pre-incubated with sodium tanshinone IIA sulfonate (STS, 25 μM) or the broad-spectrum cysteine protease inhibitor N-ethylmaleimide (NEM, 10 μM) for 15 min at room temperature prior to substrate addition. Background fluorescence was subtracted, and enzyme activity was quantified as the slope of fluorescence increase over time (RFU/min).

### 2.9. Statistical Analysis

All experiments were performed in at least three independent replicates. Data are presented as mean ± standard error of the mean (SEM). For comparisons across groups, multiple unpaired *t*-tests were performed with correction for multiple comparisons where appropriate. *p* values ≤ 0.05 were considered statistically significant. All statistical analyses and graphing were performed using GraphPad Prism (version 10.0; GraphPad Software, Boston, MA, USA).

## 3. Results

### 3.1. Usp31 Exhibits Sustained Upregulation Following Oxygen-Glucose Deprivation and Reoxygenation in Cardiomyocytes

**Figure 1:**
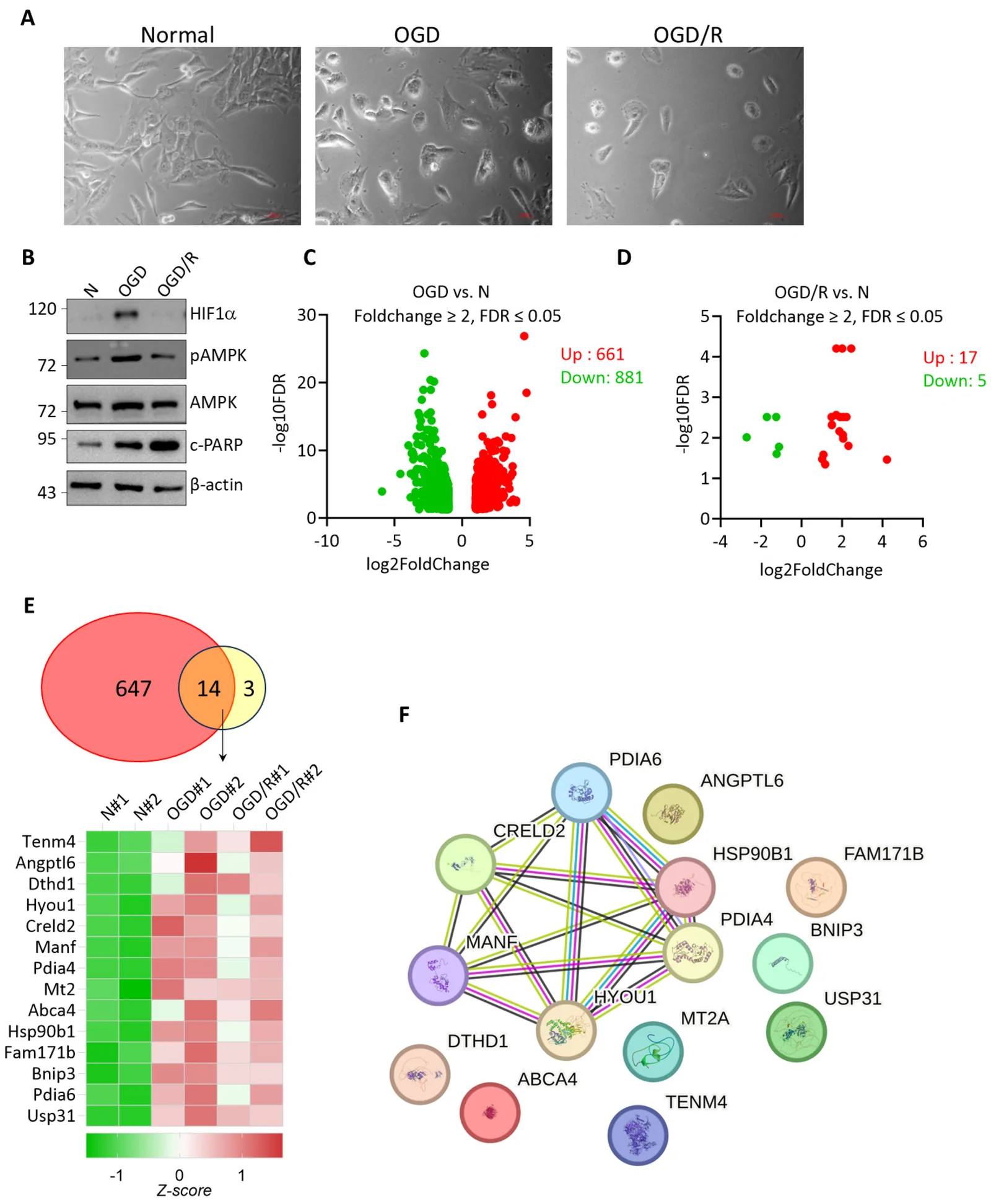
**Transcriptomic profiling identifies stress-response genes persistently induced during OGD/R in cardiomyocytes.** (**A**) Representative morphological images of HL-1 cells under different treatment conditions using the conventional 20% O_2_-based OGD/R protocol. N, normal culture condition under 20% O_2_ with 25 mM glucose; OGD, oxygen-glucose deprivation under 1% O_2_ in glucose-free medium for 6 h; OGD/R, OGD for 6 h followed by reoxygenation under 20% O_2_ with glucose replenishment for 6 h (scale bar 50 µm). (**B**) Immunoblot analysis of the indicated markers in HL-1 cells under normal, OGD, and OGD/R conditions using the conventional 20% O_2_-based OGD/R protocol. HIF1α was used as a marker of hypoxic stress, and p-AMPK was used as a marker of metabolic stress. (**C**,**D**) Volcano plot analysis of differentially expressed genes (DEGs) between OGD and normal conditions (**C**) and between OGD/R and normal conditions (**D**). Genes with absolute fold change ≥ 2 and FDR-adjusted *p* value ≤ 0.05 were considered significantly differentially expressed. Red dots indicate significantly upregulated genes, and green dots indicate significantly downregulated genes. (**E**) Venn diagram and heatmap illustrating the overlap of genes upregulated under both OGD and OGD/R conditions. (**F**) Protein-protein interaction (PPI) network analysis using STRING for the 14 genes commonly upregulated under both OGD and OGD/R conditions. In RNA-seq sample labels, #1 and #2 denote independent biological replicates.

To elucidate the molecular regulatory network underlying myocardial injury induced by oxygen-glucose deprivation (OGD) and subsequent reoxygenation (OGD/R), and to identify potential candidate stress-responsive regulators, we performed RNA sequencing on mouse cardiomyocyte HL-1 cells subjected to OGD for 6 h or OGD followed by 6 h of reoxygenation and glucose replenishment, alongside normal controls (Figure 1A,B and Figure S1). Differentially expressed genes (DEGs) were identified using thresholds of fold change ≥ 2 and FDR ≤ 0.05. Compared with normal conditions, OGD induced 661 upregulated and 881 downregulated genes (Figure 1C). In contrast, the OGD/R group exhibited substantially fewer genes meeting the predefined differential-expression criteria relative to controls, with 17 upregulated and 5 downregulated genes relative to controls (Figure 1D), indicating a distinct transcriptional state after reoxygenation rather than a simple continuation of the OGD response. Nonetheless, myocardial ischemia-reperfusion is known to elicit sustained cellular damage, which may be attributed to genes persistently dysregulated after OGD/R. Comparative analysis identified 14 genes upregulated under both OGD and OGD/R conditions (Figure 1E,F). Several of these, including Pdia4&6 [16,17], Hyou1 [18], Hsp90b1 [19], Manf [20], Bnip3 [21], and Mt2 [22], are well-established mediators of cellular stress responses, underscoring the biological relevance of our transcriptomic findings.

**Figure 2:**
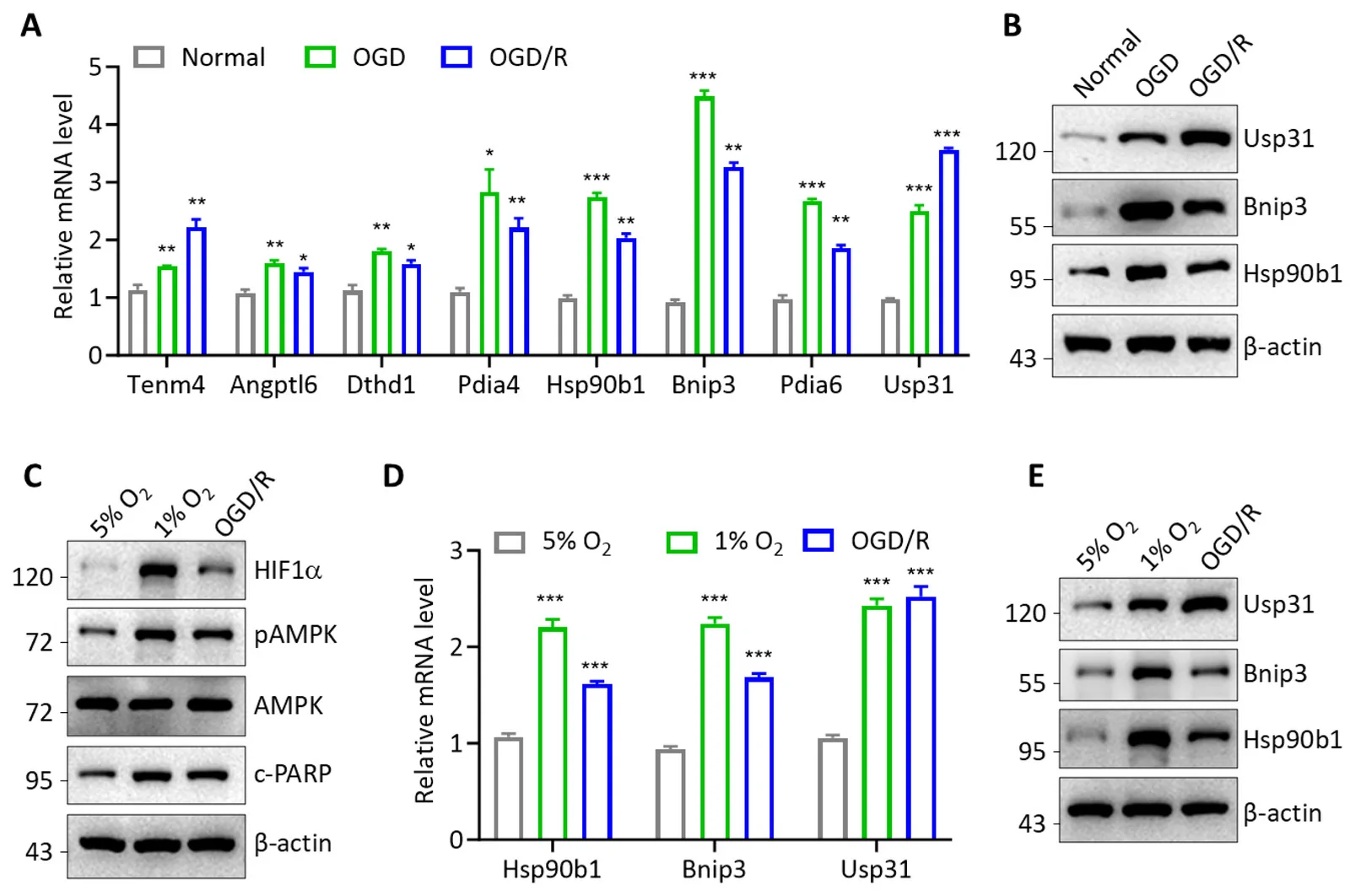
**Usp31 is persistently induced during OGD/R under conventional and physiologic oxygen-based culture conditions.** (**A**) qRT-PCR validation of representative genes persistently upregulated under both OGD and OGD/R conditions in HL-1 cells using the conventional 20% O_2_-based OGD/R protocol. Data are presented as mean ± SEM. *, *p* < 0.05; **, *p* < 0.01; ***, *p* < 0.001. (**B**) Immunoblot analysis of the indicated protein levels in HL-1 cells under normal, OGD, and OGD/R conditions using the conventional 20% O_2_-based OGD/R protocol. β-actin was used as the loading control. (**C**) Immunoblot analysis of the indicated markers in HL-1 cells using the physiologic 5% O_2_-based OGD/R protocol. Cells were maintained under 5% O_2_ with 25 mM glucose, subjected to OGD under 1% O_2_ in glucose-free medium for 6 h, and then reoxygenated under 5% O_2_ with glucose replenishment for 6 h. HIF1α was used as a marker of hypoxic stress, and p-AMPK was used as a marker of metabolic stress. (**D**) qRT-PCR validation of representative genes persistently upregulated under OGD and OGD/R conditions using the physiologic 5% O_2_-based protocol. Data are presented as mean ± SEM. ***, *p* < 0.001. (**E**) Immunoblot analysis of Usp31 and the indicated proteins in HL-1 cells under the physiologic 5% O_2_-based OGD/R protocol. β-actin was used as the loading control.

To minimize the possibility that the RNA-seq results reflected stochastic variation, we next validated the expression of these candidate transcripts by qRT-PCR. Consistently, qRT-PCR confirmed significant elevation of these genes under both OGD and OGD/R conditions (Figure 2A). Notably, Tenm4 and Usp31 exhibited sustained upregulation specifically under OGD/R. TENM4, a large transmembrane protein, has been implicated in neurodevelopment, cell adhesion, and signal transduction, with emerging roles in neurological disorders and malignancies [23]. Usp31, a member of the ubiquitin-specific protease family, functions as a canonical deubiquitinating enzyme, previously reported to target TRAF2 and p65, thereby modulating NF-κB signaling [11,13]. Given the growing interest in DUBs as therapeutic targets [24,25], Usp31 emerged as a molecule of particular interest. Immunoblotting further demonstrated that Usp31 protein levels remained elevated after reoxygenation compared to OGD alone, consistent with its transcriptional profile (Figure 2B).

Considering that the physiological oxygen tension of cardiac tissue is substantially lower than atmospheric oxygen levels and is estimated to be approximately 5% O_2_, we further modified the culture system to better approximate a physiologically relevant oxygen environment. Specifically, HL-1 cells were maintained under 5% O_2_ as the basal culture condition, subjected to OGD under the same glucose-deprivation and hypoxic conditions, and then returned to 5% O_2_ during the reoxygenation phase, while all other experimental parameters were kept unchanged (Figure 2C). Under this physiologic oxygen-based OGD/R setting, qRT-PCR analysis confirmed a similar induction pattern of Usp31 and representative stress-related genes (Figure 2D). Consistently, immunoblotting demonstrated that Usp31 protein expression was also increased after OGD/R under 5% O_2_ reoxygenation conditions (Figure 2E). These findings indicate that Usp31 upregulation is not merely a consequence of re-exposure to atmospheric oxygen, but rather represents a robust response to oxygen-glucose deprivation and subsequent reoxygenation, regardless of whether cells are recovered under conventional normoxic or physiologically relevant oxygen conditions.

To further assess whether this response was conserved in a human cardiomyocyte-derived cellular model, we performed the same OGD/R protocol in AC16 cells, consisting of 6 h OGD followed by 6 h reoxygenation. Immunoblotting showed that 6 h OGD led to marked accumulation of HIF1α and increased phosphorylation of AMPK, confirming the induction of hypoxic and metabolic stress responses. After 6 h of reoxygenation with glucose replenishment, both HIF1α and p-AMPK declined toward basal levels, indicating transition from the OGD phase to an early reoxygenation state. Notably, USP31 protein expression was also substantially increased in AC16 cells during OGD/R (Figure S2). Together, these results suggest that USP31 induction during OGD/R is observed in both mouse HL-1 cardiomyocytes and human cardiomyocyte-derived AC16 cells, supporting USP31 as a conserved stress-responsive regulatory component during oxygen-glucose deprivation and subsequent reoxygenation.

### 3.2. Usp31 Promotes Cardiomyocyte Death Following OGD/R by Sustaining NF-κB Activation

**Figure 3:**
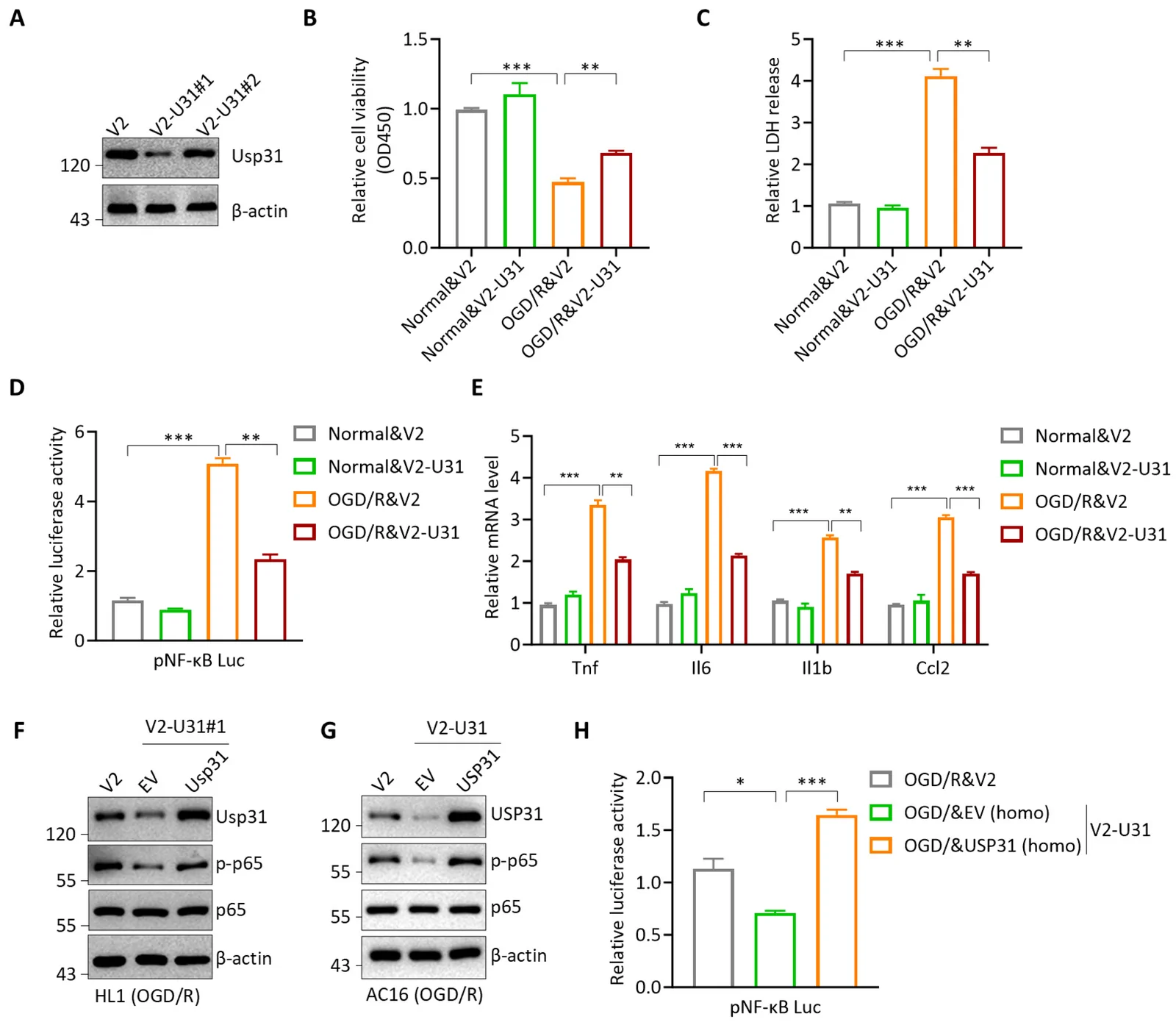
**Usp31 deficiency attenuates OGD/R-induced cardiomyocyte injury by suppressing NF-κB activation.** (**A**) Immunoblot analysis showing the knockout efficiency of two sgRNAs targeting Usp31 in HL-1 cells. U31#1 and U31#2 indicate two independent Usp31-targeting sgRNAs/clones. U31#1 showed more efficient Usp31 depletion than U31#2 and was selected for subsequent functional experiments. (**B**) CCK-8 assay showing cell viability in control and Usp31-deficient HL-1 cells under basal and OGD/R conditions. Experiments were performed using the conventional 20% O_2_-based OGD/R protocol. **, *p* < 0.01; ***, *p* < 0.001. (**C**) Quantification of LDH release in culture supernatants showing reduced membrane damage in Usp31-deficient HL-1 cells after OGD/R treatment. Experiments were performed using the conventional 20% O_2_-based OGD/R protocol. **, *p* < 0.01; ***, *p* < 0.001. (**D**) NF-κB luciferase reporter assay showing that Usp31 deficiency suppresses OGD/R-induced NF-κB transcriptional activation in HL-1 cells. Experiments were performed using the conventional 20% O_2_-based OGD/R protocol. **, *p* < 0.01; ***, *p* < 0.001. (**E**) Relative mRNA expression of NF-κB target genes, including Tnf, Il6, Il1b, and Ccl2, measured by qRT-PCR in control and Usp31-deficient HL-1 cells after OGD/R treatment. Data are presented as mean ± SEM. **, *p* < 0.01; ***, *p* < 0.001. (**F**) Immunoblot analysis of phosphorylated p65 in control, Usp31-deficient, and Usp31-rescued HL-1 cells following OGD/R treatment. Re-expression of Usp31 restored p-p65 levels in Usp31-deficient cells. (**G**) Immunoblot analysis of phosphorylated p65 in control, USP31-deficient, and USP31-rescued human cardiomyocyte-derived AC16 cells following OGD/R treatment, showing a similar rescue effect of USP31 on NF-κB pathway activation. (**H**) NF-κB luciferase reporter assay confirming that USP31 deficiency suppresses OGD/R-induced NF-κB transcriptional activation, whereas USP31 re-expression restores NF-κB activity under OGD/R conditions. *, *p* < 0.05; ***, *p* < 0.001.

To investigate the functional role of Usp31, which was persistently upregulated following OGD/R treatment in HL-1 cardiomyocytes, we first established a CRISPR/Cas9-mediated Usp31-deficient cell model. Among the two sgRNA-targeting sequences tested, U31#1 showed more efficient depletion of Usp31 than U31#2 (Figure 3A). Because U31#2 produced insufficient depletion for reliable functional analysis, U31#1 was selected for subsequent experiments.

CCK-8 assays showed no significant difference in cell viability between Usp31-deficient and control HL-1 cells under basal culture conditions. However, after 6 h of OGD followed by 6 h of reoxygenation, Usp31 deficiency markedly improved cell viability (Figure 3B). Consistently, LDH release, an indicator of membrane damage and cell injury, was significantly reduced in the culture supernatant of Usp31-deficient cells compared with control cells after OGD/R treatment (Figure 3C), suggesting that loss of Usp31 alleviates OGD/R-induced cardiomyocyte injury.

Because Usp31 has previously been implicated in NF-κB pathway regulation, and because sustained inflammatory signaling contributes to cardiomyocyte damage during OGD/R, we next examined NF-κB pathway activity. Dual-luciferase reporter assays showed that NF-κB transcriptional activity was robustly induced after OGD/R, whereas Usp31 deficiency significantly suppressed this activation (Figure 3D). Further analysis of canonical NF-κB target genes demonstrated that Tnf, Il6, Il1b, and Ccl2 were upregulated in response to OGD/R, while Usp31 depletion markedly attenuated their induction (Figure 3E).

To further verify whether Usp31 directly contributes to NF-κB pathway activation under OGD/R conditions, we re-expressed Usp31 in Usp31-deficient HL-1 cells. Following OGD/R treatment, restoration of Usp31 markedly increased the level of phosphorylated p65 (p-p65), indicating reactivation of NF-κB signaling (Figure 3F). Similar results were observed in human cardiomyocyte-derived AC16 cells, in which USP31 re-expression restored p-p65 levels in USP31-deficient cells after OGD/R treatment (Figure 3G). In addition, dual-luciferase reporter assays further confirmed that USP31 deficiency significantly inhibited OGD/R-induced NF-κB transcriptional activation, whereas USP31 re-expression substantially restored NF-κB activity (Figure 3H).

Collectively, these findings suggest that Usp31 contributes to OGD/R-induced cardiomyocyte injury by promoting NF-κB pathway activation. Importantly, the rescue experiments in both mouse HL-1 cells and human cardiomyocyte-derived AC16 cells support a conserved role of Usp31 in regulating NF-κB-associated inflammatory signaling during oxygen-glucose deprivation and subsequent reoxygenation.

### 3.3. Sodium Tanshinone IIA Sulfonate (STS) Inhibits the Deubiquitinase Activity of Usp31

**Figure 4:**
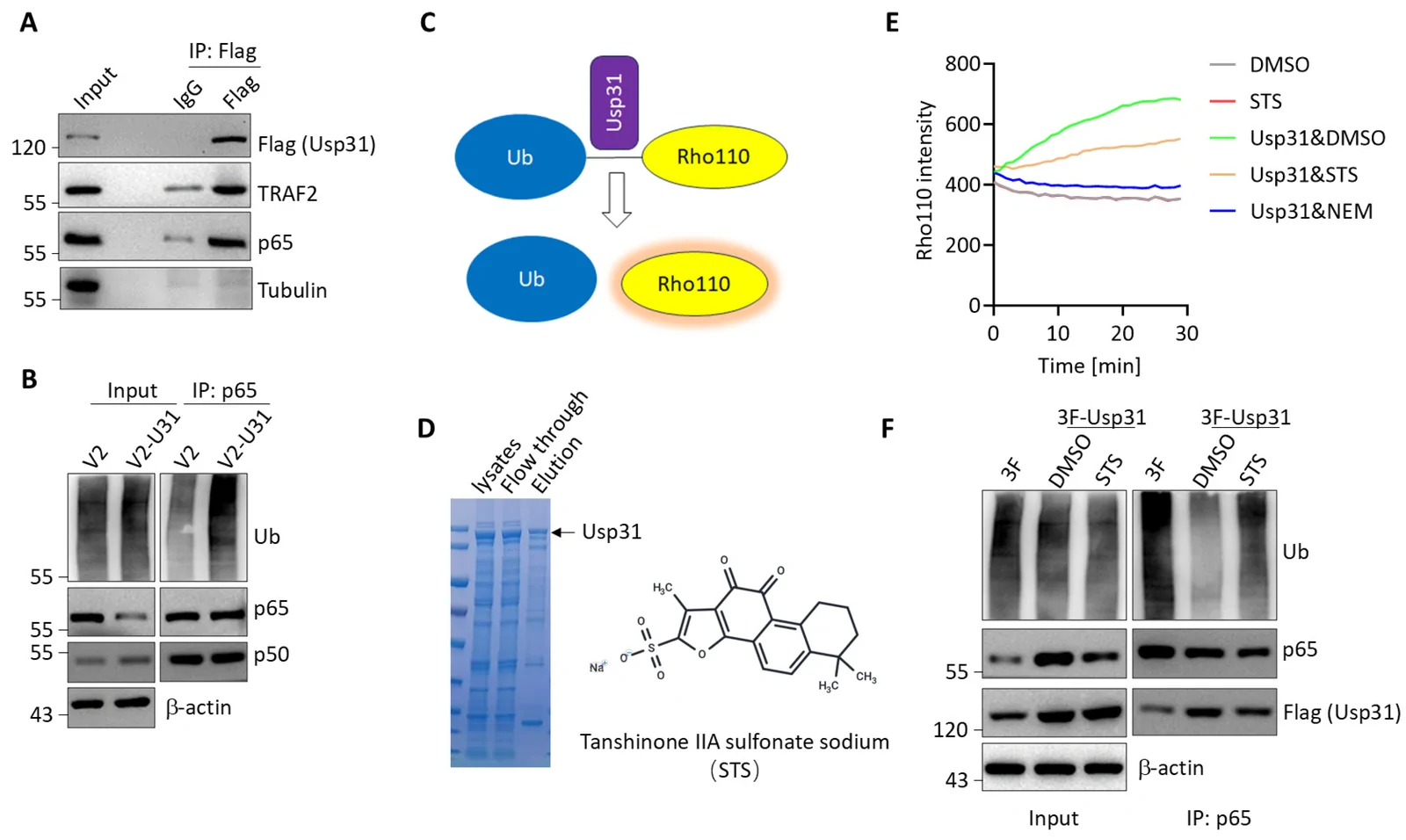
**Usp31 regulates p65 ubiquitination, and STS inhibits recombinant Usp31 enzymatic activity in vitro.** (**A**) Co-immunoprecipitation assay showing the interaction between Flag-tagged Usp31 and endogenous TRAF2 and p65 in HL-1 cells following OGD/R treatment. (**B**) Immunoprecipitation of p65 followed by immunoblot analysis of the indicated proteins in control and Usp31-deficient HL-1 cells under OGD/R conditions. p50 was used as a binding control for p65. (**C**) Schematic diagram of the Ub-Rho110-based high-throughput screening assay used to identify compounds that inhibit Usp31 deubiquitinase activity. (**D**) Coomassie brilliant blue staining of purified recombinant Usp31 protein expressed in *E. coli* and the chemical structure of sodium tanshinone IIA sulfonate (STS). (**E**) Ub-Rho110-based fluorogenic assay showing that STS inhibits the enzymatic activity of recombinant Usp31 in vitro. N-ethylmaleimide (NEM) was used as a positive control for inhibition of deubiquitinase activity. (**F**) Immunoprecipitation of p65 followed by immunoblotting for ubiquitin in HL-1 cells under OGD/R conditions, with or without Usp31 overexpression. Usp31-overexpressing cells were treated with either vehicle or STS during OGD/R treatment to assess the effect of STS on Usp31-associated p65 deubiquitination.

Previous studies have shown that Usp31 promotes NF-κB pathway activation by stabilizing TRAF2 and p65. To determine whether Usp31 interacts with these molecules in cardiomyocytes, we first expressed Flag-tagged Usp31 in HL-1 cells and subjected them to OGD/R treatment. Co-immunoprecipitation assays revealed that Usp31 physically associates with both TRAF2 and p65 under OGD/R conditions (Figure 4A). We then examined the ubiquitination status of p65 in Usp31-knockout and control HL-1 cells following OGD/R. Notably, p65 ubiquitination was markedly increased in the absence of Usp31 (Figure 4B), indicating that sustained Usp31 elevation during OGD/R promotes NF-κB activation by preventing p65 degradation.

These findings suggest that Usp31 may serve as a potential therapeutic target in myocardial infarction. To identify pharmacological inhibitors of Usp31, we performed high-throughput screening using a commercial drug library and an established Ub-Rho110-based in vitro assay (Figure 4C). Recombinant Usp31 protein was purified from *E. coli* and used for activity screening (Figure 4D). Among the candidate compounds identified from the screening assay, sodium tanshinone IIA sulfonate (STS), a clinically used cardiovascular compound, showed inhibitory activity against recombinant Usp31 in the Ub-Rho110-based deubiquitinase assay (Figure 4E). Further concentration-response analysis demonstrated that STS inhibited Usp31 enzymatic activity with an IC50 value of 20.2 μM (Figure S3). These findings suggest that Usp31-associated deubiquitination may represent one intracellular signaling event modulated by STS, although additional studies are required to determine the selectivity of STS toward Usp31 and to define whether Usp31 is a major cellular target of STS.

### 3.4. STS Regulates Usp31-Associated Signaling to Restrain NF-κB–Driven Inflammation and Cell Death in Cardiomyocytes

**Figure 5:**
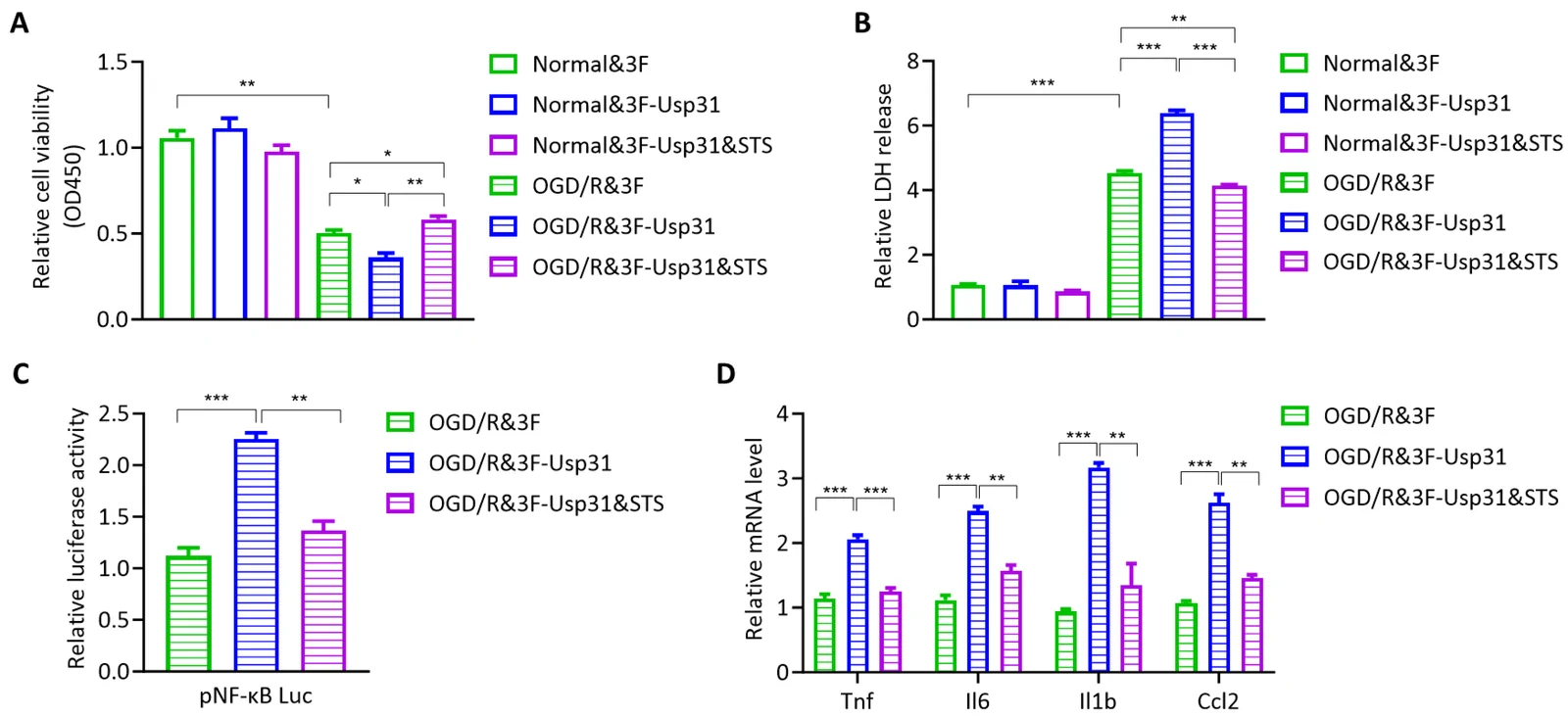
**STS attenuates Usp31-associated NF-κB activation and cardiomyocyte injury during OGD/R.** (**A**) CCK-8 assay showing that STS treatment improves cell viability in Usp31-overexpressing HL-1 cells under OGD/R conditions. Experiments were performed using the conventional 20% O_2_-based OGD/R protocol. *, *p* < 0.05; **, *p* < 0.01. (**B**) LDH release assay showing that STS treatment reduces membrane damage in Usp31-overexpressing HL-1 cells under OGD/R conditions. **, *p* < 0.01; ***, *p* < 0.001. (**C**) NF-κB luciferase reporter assay demonstrating that STS attenuates NF-κB activation induced by Usp31 overexpression during OGD/R. **, *p* < 0.01; ***, *p* < 0.001. (**D**) qRT-PCR analysis showing that STS reduces the mRNA expression of NF-κB target genes, including Tnf, Il6, Il1b, and Ccl2, in Usp31-overexpressing HL-1 cells under OGD/R conditions. Data are presented as mean ± SEM. **, *p* < 0.01; ***, *p* < 0.001.

To investigate whether STS modulates the cellular effects of Usp31, we treated HL-1 cells overexpressing Usp31 or vector control with STS under both normal and OGD/R conditions. CCK-8 assays revealed that STS treatment significantly rescued the reduction in cell viability caused by Usp31 overexpression (Figure 5A). Consistently, LDH release into the culture medium was markedly decreased following STS treatment, indicating reduced cellular injury (Figure 5B). Furthermore, dual-luciferase reporter assays demonstrated that STS effectively suppressed NF-κB transcriptional activity enhanced by Usp31 overexpression (Figure 5C), along with a significant reduction in the expression of NF-κB target genes (Figure 5D).

Taken together, these findings indicate that sustained elevation of Usp31 during OGD/R promotes cardiomyocyte injury by amplifying NF-κB-mediated inflammatory responses, and that STS mitigates these effects at least in part by suppressing Usp31-associated signaling. Thus, Usp31 is a functional regulatory node involved in the cellular response to STS treatment under cardiomyocyte stress conditions.

## 4. Discussion

Myocardial ischemia-reperfusion (I/R) injury remains a major challenge in cardiovascular disease, primarily due to excessive oxidative stress, inflammatory activation, and subsequent cardiomyocyte dysfunction [1]. Among the signaling pathways involved, NF-κB has been well recognized as a major mediator of inflammation in ischemic hearts [26]. However, the upstream regulatory mechanisms responsible for sustaining NF-κB activation during and after ischemic stress remain incompletely understood. In this study, we identified Usp31, a member of the ubiquitin-specific protease (USP) family, as a previously uncharacterized candidate regulator involved in NF-κB-associated inflammatory responses during ischemic stress.

Mechanistically, we demonstrate that Usp31 interacts with NF-κB key mediators including TRAF2 and p65, removing ubiquitin chains and thereby stabilizing p65 to maintain NF-κB transcriptional activity. Depletion of Usp31 markedly attenuated NF-κB target gene expression and reduced cardiomyocyte injury, functionally validating its importance. These findings place Usp31 as a key component of the ubiquitin-editing machinery that modulates inflammatory signaling under ischemic stress.

Although the role of deubiquitinating enzymes in ischemic injury is beginning to be appreciated, current literature remains limited. For instance, USP10 [27] and USP13 [28] have been implicated in regulating autophagy and oxidative stress during cardiac injury, but few studies have addressed DUB-mediated control of NF-κB signaling in the context of OGD/R. To our knowledge, this is the first report to characterize Usp31 as a modulator of NF-κB–dependent inflammation in ischemic cardiomyocytes.

From a translational perspective, we provide evidence that sodium tanshinone IIA sulfonate (STS), a water-soluble derivative of tanshinone IIA [29], can directly inhibit Usp31 activity. STS enhanced p65 ubiquitination, suppressed NF-κB signaling, and reduced cardiomyocyte damage under ischemic stress. These data not only support a novel intracellular target of STS but also suggest its repurposing potential as an anti-inflammatory therapy for myocardial I/R injury.

The physiological relevance of our findings should be interpreted within the limitations of the experimental models used. Although HL-1 cardiomyocytes provide a widely used and experimentally tractable model for investigating cardiomyocyte responses to ischemic stress, they cannot fully reproduce the complex microenvironment of myocardial I/R injury in vivo. To improve the translational relevance of our findings, key observations were further validated in human cardiomyocyte-derived AC16 cells, supporting that Usp31 regulation is not restricted to a single mouse-derived cardiomyocyte model. Nevertheless, future studies using primary cardiomyocytes, animal myocardial I/R models, and human myocardial samples will be necessary to further establish the physiological significance of Usp31 regulation in cardiac injury.

Several additional limitations should be acknowledged. First, RNA-seq analysis was performed with a limited number of biological replicates, which may influence statistical power and sensitivity for detecting subtle transcriptional changes. Although PCA and differential expression analysis supported dynamic transcriptional changes during OGD/R, larger sequencing cohorts will be required to further define the transcriptional network regulated by Usp31. Second, the 6 h OGD followed by 6 h reoxygenation protocol was selected based on preliminary optimization experiments. Prolonged OGD exposure resulted in excessive cell death, which may interfere with mechanistic investigations, whereas the selected condition provided a reproducible injury model suitable for studying early stress-responsive signaling events. Future studies using extended reperfusion periods will be valuable for investigating long-term consequences, including cardiac remodeling and fibrosis. Furthermore, the upstream signals responsible for Usp31 induction during OGD/R remain unknown and warrant further exploration.

In conclusion, our study identifies Usp31 as a previously uncharacterized regulatory component involved in NF-κB-associated inflammatory responses in ischemic cardiomyocytes. By integrating transcriptomic analysis, genetic manipulation, biochemical evaluation, and pharmacological intervention, we provide evidence that Usp31 contributes to cardiomyocyte responses to ischemic stress and represents a potential target for further investigation. These findings provide new insights into ubiquitin-dependent regulation of ischemic injury and highlight Usp31-associated signaling as a potential mechanism underlying the pharmacological effects of STS.

## Data Availability

The data presented in this study are available on request from the corresponding authors due to ongoing related analyses and institutional data-management considerations.

## Supplementary Materials

The following supporting information can be downloaded at: https://www.mdpi.com/article/10.3390/biomedicines14081812/s1, Figure S1: Principal component analysis (PCA) of RNA-seq samples; Figure S2: Validation of the OGD/R model and USP31 induction in AC16 cells; Figure S3: Dose-response analysis of STS inhibition of recombinant USP31 activity; Table S1: Primer sequences.
