# Supplementary material for: Usp31 Promotes Cardiomyocyte Injury Under Ischemic Stress and Is Inhibited by Sodium Tanshinone IIA Sulfonate
Source: Biomedicines. 2026 Aug 12;14(8):1812. doi: 10.3390/biomedicines14081812 (PMC13509399; doi:10.3390/biomedicines14081812)
Supplement: Supplementary file 1 [file biomedicines-14-01812-s001.zip › biomedicines-4373528-supplementary.pdf]

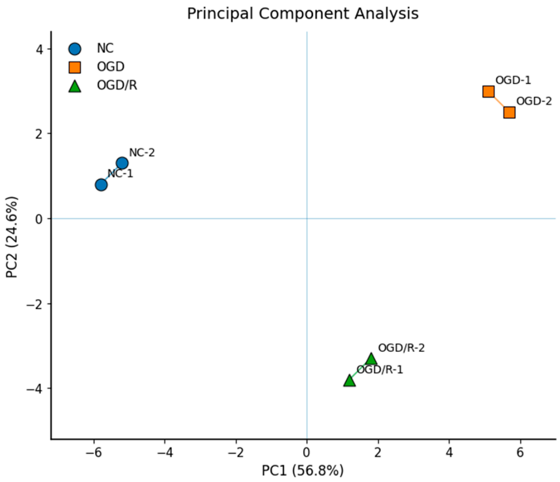

Supplementary Figure S1 Principal component analysis (PCA) of RNA-seq samples

Principal component analysis of transcriptomic profiles under normal, OGD, and OGD/R conditions. Principal component analysis (PCA) was performed to visualize the overall differences in gene-expression profiles among HL-1 cells cultured under normal conditions (NC), subjected to oxygen–glucose deprivation (OGD), or exposed to OGD followed by reoxygenation and glucose replenishment (OGD/R). Each point represents an independent biological sample, with two replicates included per group. PC1 and PC2 explained 56.8% and 24.6% of the total variance, respectively. Biological replicates within each group clustered closely, whereas the three experimental groups were clearly separated, indicating distinct transcriptomic profiles under NC, OGD, and OGD/R conditions.

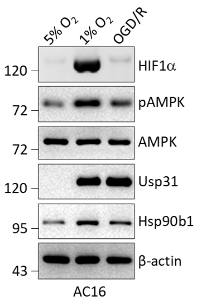

Supplementary Figure S2 Validation of the OGD/R model and USP31 induction in AC16 cells

Representative Immunoblot analysis of HIF1α, p-AMPK, and USP31 in AC16 cells subjected to OGD for 6 h followed by 6 h reoxygenation. （n=3）.

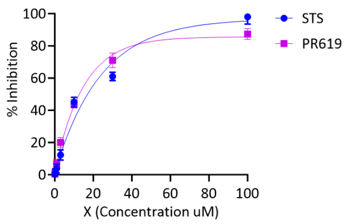

Supplementary Figure S3 Dose-response analysis of STS inhibition of recombinant USP31 activity

Concentration-dependent inhibition of recombinant Usp31 activity by STS

Ub-Rho110-based enzymatic assay showing concentration-dependent inhibition of recombinant Usp31 activity by STS in vitro. The IC50 value of STS against recombinant Usp31 was calculated as 20.2 μM.

Table S1 primers sequences

Gene	Forward Primer	Reverse Primer
Tnf	CCCTCACACTCAGATCATCTTCT	GCTACGACGTGGGCTACAG
Il6	TAGTCCTTCCTACCCCAATTTCC	TTGGTCCTTAGCCACTCCTTC
Il1b	GCAACTGTTCCTGAACTCAACT	ATCTTTTGGGGTCCGTCAACT
Ccl2	TTAAAAACCTGGATCGGAACCAA	GCATTAGCTTCAGATTTACGGGT
Hsp90b1	GAACTTGAGGAGGTGCTGGA	TGTCTCGTCAGGGATGATGA
Bnip3	TTGAAGGTCGCCGGTTCT	TTGTGGGATGGTGCTGTTG
Pdia6	CTGTGGTGATCGGTGTGGAG	AGCCAGTCACCATGTCCTTC
Usp31	AGGGAGGAGTGTTGATGGAG	CTGTAGCAGCAGTTGGTGTG
Tenm4	GGAATCATCTCCACTTTGCTGGG	GGTTGATGGCTAAGTCTGTGGG
Angptl6	TCCATCAGCTGCACTGTCTT	TGCAGAGTGTGTCCAGTTCC

Dthd1	TGCCTGTGAGAGTGCTGAAA	CCCAACACTTTGGTGAGTGG
Pdia4	ATGAGTGCTTGTGGAAGACG	CGTGGTAGTCAGGCTTCTGA
Gapdh	AGGTCGGTGTGAACGGATTTG	TGTAGACCATGTAGTTGAGGTCA
